# A Novel Role of Irbesartan in Gastroprotection against Indomethacin-Induced Gastric Injury in Rats: Targeting DDAH/ADMA and EGFR/ERK Signaling

**DOI:** 10.1038/s41598-018-22727-6

**Published:** 2018-03-09

**Authors:** Nancy N. Shahin, Noha F. Abdelkader, Marwa M. Safar

**Affiliations:** 1 Department of Biochemistry, Faculty of Pharmacy, Cairo University, Cairo, Egypt; 2 Department of Pharmacology and Toxicology, Faculty of Pharmacy, Cairo University, Cairo, Egypt; 3 Department of Pharmacology and Biochemistry, Faculty of Pharmacy, the British University in Egypt, Cairo, Egypt

## Abstract

The advent of angiotensin II type 1 receptor blockers (ARBs) as intriguing gastroprotective candidates and the superior pharmacokinetics and pharmacodynamics displayed by irbesartan compared to many other ARBs raised the interest to investigate its gastroprotective potential in a rat model of gastric injury. Irbesartan (50 mg/Kg) was orally administered to male Wistar rats once daily for 14 days; thereafter gastric injury was induced by indomethacin (60 mg/Kg, p.o). Irbesartan reduced gastric ulcer index, gastric acidity, and ameliorated indomethacin-induced gastric mucosal apoptotic and inflammatory aberrations, as demonstrated by hampering caspase-3, prostaglandin E_2_ and tumor necrosis factor-alpha levels and cyclooxygenase-2 mRNA expression. This ARB increased mucosal dimethylarginine dimethylaminohydrolase-1 (DDAH-1) gene expression and decreased elevated levels of matrix metalloproteinase-9, asymmetric dimethylarginine (ADMA), epidermal growth factor receptor (EGFR) mRNA and phosphorylated extracellular signal-regulated kinase 1 and 2 (pERK1/2). Histopathological evaluation corroborated biochemical findings. Overall efficacy of irbesartan was comparable to ranitidine, the widely used H_2_ receptor blocker. In conclusion, irbesartan exerts significant gastroprotection against indomethacin-induced mucosal damage via acid-inhibitory, anti-inflammatory, anti-apoptotic and extracellular matrix remodeling mechanisms that are probably mediated, at least partly, by down-regulating DDAH/ADMA and EGFR/ERK1/2 signaling.

## Introduction

Peptic ulcer is one of the most common gastrointestinal disorders with 4–5% prevalence in the human society^1^. Long-term use of non-steroidal anti-inflammatory drugs (NSAIDs) is the second most common cause of peptic ulcer disease after *Helicobacter pylori* (*H. pylori*) infection^2^. Patients with cardiovascular disease and hypertension are at increased risk of developing NSAID-induced gastrointestinal tract complications; there are several reports linking hypertension and vascular damage to gastric mucosal damage^3^. More than half of the population older than 65 years of age suffer from hypertension^4^, and it is this elderly population that is most at risk for NSAID-induced complications^5^. Inevitably, stress causing hypertension may concomitantly predispose to gastric ulcer^6^. Taking into consideration this concordant occurrence of gastric and hypertensive diseases, in addition to the adverse effects of some antihypertensive drugs on the gastric mucosa^7^, it would be worthwhile to introduce drugs that combine antihypertensive and gastroprotective effects.

Angiotensin II, the central product of the renin–angiotensin system, induces oxidative stress and inflammation^8^, and constricts the gastric vasculature^9^ by activating the angiotensin II type 1 (AT1) receptor^10^. Angiotensin II type 1 receptor blockers, have emerged as intriguing candidates for gastroprotection showing anti-secretory, antioxidant and anti-inflammatory effects^10,11^. They have also shown beneficial effects on healing of pre-existing gastric ulcers via enhancing gastric macro- and microcirculations as well as gastric tissue oxygenation^12^. Irbesartan is a highly selective AT1-receptor antagonist approved for treatment of hypertension. It seems to offer advantages beyond those attained by other angiotensin II receptor blockers (ARBs) owing to its unique and distinct pharmacokinetic and pharmacodynamic profiles^13,14^. Irbesartan has been recently reported to improve gastric emptying and ameliorate gastric microcirculation in diabetic rats^15^. In addition, it has been shown to exert TNF-α- and NO-modulating effects^16–18^. However, its protective potential against gastric injury has not been explored yet.

Blocking of prostaglandin synthesis through inhibition of the cyclooxygenase (COX) enzymes plays a cardinal role in the pathogenesis of NSAID-induced peptic ulcers^19^. However, there is indisputable evidence that other prostaglandin-independent mechanisms are also involved. These include generation of reactive oxygen species (ROS), initiation of lipid peroxidation and infiltration of neutrophils secondary to the production of inflammatory mediators such as tumor necrosis factor alpha (TNF-α) and leukotrienes^20–22^. Indomethacin is one of the most potent non-selective NSAIDs; however, its beneficial actions are limited by its gastrointestinal toxicity. Possessing the highest ulcerogenic potential among NSAIDs, it has been considered the drug of choice for the experimental induction of gastric ulcer^23^. Interestingly, a detrimental effect of indomethacin on the processes associated with cellular proliferation and apoptosis has been reported^24,25^. Induction of apoptosis occurs via ROS generation, cytochrome c release and activation of caspase-3^26^. As regards cell renewal, previous studies verified the involvement of epidermal growth factor receptor (EGFR) signaling in the proliferation and differentiation of gastric epithelial cells, as well as in gastric injury repair and ulcer healing. Of several mitogen-activated protein kinase (MAPK) family members, the extracellular signal-regulated kinases 1/2 (ERK1/2) serve as a major downstream effector of EGFR mediating its effects on the gastric epithelium^27,28^.

Several studies have highlighted the contribution of a number of autacoids acting in concert with prostaglandins in maintaining the gastric mucosal defense and inducing healing including nitric oxide (NO) and matrix metalloproteinases (MMPs)^19^. Mucosal NO content is significantly reduced in gastric mucosal injury models, suggesting that the decrease in local NO content might be a key factor in facilitating gastric mucosal injury^20^. However, the mechanisms responsible for the reduced NO content during gastric mucosal injury are not yet fully understood. Asymmetric dimethylarginine (ADMA) has been identified as the major endogenous inhibitor of nitric oxide synthase. There is increasing evidence that ADMA directly induces oxidative stress and apoptosis and is also involved in inflammatory reactions^29,30^. Noteworthy, ADMA has been shown to mediate gastric injury induced by ethanol, stress, *H. pylori* and indomethacin^31^. More than 90% of ADMA in rats is degraded via hydrolysis by dimethylarginine dimethylaminohydrolase-1 (DDAH-1), which therefore plays a vital role in maintaining NO bioavailability^32^. The pathogenesis of gastric ulcer is associated with remodeling of extracellular matrix (ECM) by various MMPs, a family of endopeptidases that selectively degrade most of the ECM components including collagen, and other structural molecules of the gastric mucosa^23^.

The present study was, therefore, undertaken to investigate the possible gastroprotective role of irbesartan in indomethacin-induced gastric injury model in rats, in an attempt to introduce a single drug that can concomitantly control hypertension and gastric injury. Targeting gastric mucosal DDAH/ADMA and EGFR/ERK1/2 signaling, MMP-9 activation, inflammatory and apoptotic cascades by AT1 receptor blockade has been addressed.

## Materials and Methods

### Animals

A total of 114 adult male Wistar rats (180–220 g, 6–7 weeks old) obtained from the laboratory animals’ farm of the Egyptian Organization for Biological Products and Vaccines, Cairo, Egypt, were used in this study. Animals were housed at the animal facility of the Faculty of Pharmacy, Cairo University, Cairo, Egypt (12 hours light/dark cycle, humidity 60 ± 10%, and temperature 25 ± 2 °C). Access to food and water throughout the experimental period was allowed *ad libitum*. All the animal protocols were in accordance with the Guide for the Care and Use of Laboratory Animals published by the US National Institutes of Health (NIH Publication No. 85–23, revised 1996) and were approved by the Ethical Committee for Animal Experimentation at Faculty of Pharmacy, Cairo University (Permit number: BC 1516).

### Drugs and Chemicals

Irbesartan was obtained from Sanofi (Cairo, Egypt), indomethacin was obtained from Sigma-Aldrich Chemical Co. (St. Louis, MO, USA) and ranitidine hydrochloride was obtained from GlaxoSmithKline (Cairo, Egypt). Other chemicals were of analytical grade.

### Experimental design

Animals were randomly allocated into 5 groups (n = 18): Group I (Normal): normal rats which served as a control group, Group II (Irb): irbesartan-treated rats, Group III (Ind): indomethacin-treated rats, Group IV (Ind + Ran): indomethacin-treated rats pretreated with the H_2_ receptor antagonist, ranitidine, as a reference anti-ulcer drug, and Group V (Ind + Irb): indomethacin-treated rats pretreated with irbesartan.

Groups I (Normal) and III (Ind) received daily oral appropriate volumes of saline for 14 days. Animals in groups II (Irb) and V (Ind + Irb) received daily oral doses of irbesartan (50 mg/Kg) suspended in saline for 14 days; this dose was selected according to a pilot experiment as well as previous investigations^16,17^. Rats in group IV (Ind + Ran) were administered ranitidine orally once daily at a dose of 50 mg/Kg for 14 days^33^. Rats were fasted but had free access to water for the last 24 hours of the experimental period. Following the 24 hours’ fasting period, indomethacin, suspended in 1% tween 80, was given to each animal in groups III, IV and V at a single oral dose of 60 mg/Kg to induce gastric injury^34^. Rats in groups I and II received the vehicle (1% tween 80) orally instead of indomethacin. Six hours after indomethacin administration, animals were sacrificed by decapitation under anesthesia.

In the pilot experiment, 24 rats were randomly allocated into four groups (n = 6). Group I (Normal), group II (Ind), group III (Ind + Irb 25), and group IV (Ind + Irb 50) were treated as previously described. These 2 doses of Irb were selected based on previous studies^35,36^. After the end of the experiment, the ulcer index was determined and the dose 50 mg/Kg was selected for the completion of the main study.

### Determination of gastric acidity

For the determination of gastric acidity, 6 rats from each group were subjected to pyloric ligation before induction of gastric injury. For each rat, the abdomen was incised and the pylorus was ligated under anesthesia. Indomethacin was given orally immediately after pyloric ligation. Control rats were given 1% tween 80 instead of indomethacin. Rats were sacrificed by decapitation 6 hours following indomethacin (or tween 80) administration. Their stomachs were excised following ligature of the oesophocardiac junction, washed with saline, dried between filter paper and opened along the greater curvature. The gastric juice was drained into a centrifuge tube through a funnel, centrifuged at 600 × g^37,38^ and used for the determination of gastric acidity.

Titrable acidity was determined as described by Grossman (1963). 0.2 ml of the supernatant of the gastric juice was titrated against 0.01 N sodium hydroxide using an end point of pH 7 as determined colorimetrically with phenol red indicator. Units were expressed as milliequivalents per liter (mEq/L). Titrable acidity was calculated according to Equation 1:

1 $${\rm{Titrable}}\,{\rm{Acidity}}\,({\rm{mEq}}/{\rm{L}})=\frac{{\rm{Volume}}\,({\rm{L}})\,{\rm{of}}\,0.01\,{\rm{N}}\,{\rm{NaOH}}\,{\rm{used}}\,{\rm{for}}\,{\rm{titration}}\times {\rm{1000}}}{{\rm{Volume}}\,({\rm{L}})\,{\rm{of}}\,{\rm{gastric}}\,{\rm{juice}}\,{\rm{taken}}\,{\rm{for}}\,{\rm{titration}}\times {\rm{100}}}$$

### Assessment of gross mucosal damage

Immediately after decapitation, stomachs were removed, opened along the greater curvature, washed with ice-cold saline, blotted dry between filter paper and pinned flat on a cardboard to be subjected to gross lesions evaluation.

#### Determination of ulcer index

The length of each lesion along its greatest diameter was measured and the sum of lengths per stomach was expressed as ulcer index (mm)^39^.

#### Determination of preventive index

The preventive index of each drug is the percentage inhibition of gastric mucosal damage produced by such drug^40^. It was calculated according to Equation 2:

2 $${\rm{Preventive}}\,{\rm{index}}=100-[\frac{{\rm{ulcer}}\,{\rm{index}}\,{\rm{of}}\,{\rm{treated}}\,{\rm{group}}}{{\rm{ulcer}}\,{\rm{index}}\,{\rm{of}}\,{\rm{control}}\,{\rm{group}}}\times {\rm{100}}]$$

### Tissue sampling

After the evaluation of the ulcer index, the gastric mucosae of 6 stomachs from each group were scraped off, weighed and each was divided into 2 portions. One portion was used for DDAH-1, EGFR, COX-2 and caspase-3 gene expression analyses. The other portion was homogenized in ice-cold phosphate buffered saline (0.02 M, pH 7.2) to obtain 10% aqueous homogenates. The resulting homogenate was centrifuged at 5000 × g for 30 minutes, and the supernatant was used for the determination of asymmetric dimethylarginine (ADMA), prostaglandin E_2_ (PGE_2_), tumor necrosis factor-alpha (TNF-α), interleukin-4 (IL-4) and phosphorylated ERK1/2 (pERK1/2) levels. In addition, 6 stomachs from each group were fixed in 10% formalin/saline for histopathological examination and immunohistochemical evaluation of matrix metalloproteinase-9 (MMP-9).

### Gene expression analyses

#### Isolation of total RNA

Total RNA extraction from gastric mucosa was done using Trizol (Invitrogen, Carlsbad, CA, USA) according to the manufacturer’s instructions and the product of extraction was stored at −80 °C. The concentration and purity of RNA were determined spectrophotometrically by the 260/280 nm ratio which ranged between 1.8 and 2.1. Additionally, integrity was assured with ethidium bromide-stain analysis of 28 S and 18 S bands by formaldehyde-containing agarose gel electrophoresis.

#### Reverse transcription reaction

The isolated total RNA was reverse-transcribed into complementary DNA (cDNA) using the High Capacity cDNA Reverse Transcription Kit (Applied Biosystems, Foster City, CA) according to the manufacturer’s instructions and all products were stored at −20 °C.

#### Real-time quantitative polymerase chain reaction (qPCR)

The expression levels of DDAH-1, EGFR, COX-2 and caspase-3 genes were analyzed by qPCR using the SYBR Green PCR Master MIX (Applied Biosystems, CA, USA) with the ABI PRISM 7000 sequence detection system (Applied Biosystems, Foster City, CA, USA) and relative quantification software (Applied Biosystems, Foster City, CA, USA). The sequences of the primers used are listed in Table 1. Glyceraldehyde-3-phosphate dehydrogenase (GAPDH) was used as the housekeeping gene. As a relative quantitation, fold changes were calculated following the 2^−∆∆Ct^ method. For each sample, the Ct value of target gene mRNA was normalized against the GAPDH endogenous control as ∆ CT (∆Ct = Ct_target gene_ − Ct_GAPDH_). The fold change of the target gene mRNA in the experimental sample relative to control sample was determined by 2^−∆∆Ct^, where ∆∆CT = ∆Ct_Experimental_ − ∆Ct_Control_.

**Table 1:** Primer sequences used for real time-PCR.

Gene	Primer Sequence	Accession number
Dimethylarginine dimethylaminohydrolase-1 (DDAH-1)	F: 5′-AGGCTGATGATGGCTCTGTA-3′ R: 5′-ATCCAGAGTTCGAGACCTTG-3′	NM_022297.2
Epidermal growth factor receptor (EGFR)	F: 5′-AGTGGTCCTTGGAAACTTGG-3′ R: 5′-GTTGACATCCATCTGGTACG-3′	NM_031507.1
Cyclooxygenase-2 (COX-2)	F: 5′-TGGTGCCGGGTCTGATGATG-3′ R: 5′-GCAATGCGGTTCTGATACTG-3′	NM_017232.3
Caspase-3	F: 5′-GGTATTGAGACAGACAGTGG-3′ R: 5′-CATGGGATCTGTTTCTTTGC-3′	NM_012922.2
GAPDH	F: 5′-AAGCTGGTCATCAATGGGAAAC-3′ R: 5′-GAAGACGCCAGTAGACTCCACG-3′	NM_017008.4

### Evaluation of gastric inflammatory parameters

Gastric mucosal ADMA, PGE_2_, TNF-α and IL-4 levels were assayed by rat enzyme-linked immunosorbent assay (ELISA) kits supplied by USCN Life Science (Wuhan, China), Cayman Chemical (MI, USA), Immuno-Biological Laboratories-IBL (Gunma, Japan) and R&D Systems (Minneapolis, MN, USA), respectively according to the manufacturers’ instructions.

### Determination of pERK1/2 level

Gastric mucosal pERK1/2 level was estimated using the TiterZyme® CLIA phospho-ERK1/2 chemiluminescence enzyme immunometric assay kit supplied by Assay Designs (Ann Arbor, USA) according to the manufacturer’s protocol.

### Histopathological investigation

Ten percent formalin-fixed gastric mucosal tissues, from 6 stomachs/group, were embedded in paraffin after gradient dehydration. Paraffin wax tissue blocks were cut at 4 microns thickness by slidge microtome. The obtained tissue sections were deparaffinized and stained by hematoxylin and eosin (H&E) for histopathological examination through the light microscope^41^. Specimens were scored under light microscopy using a scoring system that includes the graded assessment of gastric mucosal injury, neutrophil infiltration and gastric hemorrhage (Table 2). A scale of 0–4 was used^42^.

**Table 2:** Histological scoring criteria.

Pathological state	Score	
Gastric mucosal injury	0	Intact
	1	Desquamation of epithelial lamina
	2	Desquamation of superficial lamina propria or 1/3 reduction of gastric glands
	3	Desquamation of middle lamina propria or 2/3 reduction of gastric glands
	4	Desquamation of lower lamina propria or >2/3 reduction of gastric glands, even exposure of submucosa
Leukocytes infiltration	0	Absent
	1	2–10/HPF
	2	11–20/HPF
	3	21–30/HPF
	4	>31/HPF
Gastric hemorrhage	0	Absent
	1	<10% of total area/LPF
	2	11–20% of total area/LPF
	3	21–30% of total area/LPF
	4	>30%

HPF: high power field; LPF: low power field.

### Immunohistochemical evaluation of MMP-9

Evaluation of gastric mucosal MMP-9 expression was performed using paraffin-embedded tissue sections of 4-μm thickness. To increase the number of antigenic sites available for binding by the antibody, sections were pretreated with 0.03% trypsin for 1 hour at 37 °C. Tissues were then placed in 3% hydrogen peroxide/methanol for 20 minutes at room temperature to block endogenous tissue peroxidase activity, followed by washing in phosphate-buffered saline. Subsequently, the tissue sections were treated with 2% bovine serum albumin for 20 minutes at room temperature. The tissue sections were then incubated with mouse anti-rat MMP-9 monoclonal antibody (MA5-14228; Thermo Scientific, Rockford, IL, USA) for 60 minutes at 37 °C. After phosphate-buffered saline washing, a secondary antibody (Dako, Copenhagen, Denmark) was applied for 60 minutes followed by the addition of horseradish peroxidase-conjugated streptavidin for 60 minutes. After 3 additional washes in phosphate-buffered saline, the immune reaction was visualized with 3,3′-diaminobenzidine (DAB) (Dako, Copenhagen, Denmark). The slides were then counterstained with hematoxylin, mounted and examined. The intensity of the immunostaining was scored according to a modification of the scoring system described by Mori *et al*.^43^ as strongly positive (3), moderately positive (2), weakly positive (1), or absent (0). The fraction of DAB-positive immunoreactive area in 6 fields/section was calculated in HPF (X400) as area percentage of immunopositive cells to the total area of the microscopic field using image analysis software (Leica QWin Plus v3; Leica Microsystems Ltd, Switzerland).

### Statistical analysis

Data are expressed as mean ± standard error of the mean (S.E.M.), and analyzed using one-way analysis of variance (ANOVA) with subsequent multiple comparisons using Tukey’s test; except for the pathological and immunostaining scores which are presented as median ± range, and the statistical variation among groups was tested by Kruskal-Wallis test followed by Dunn’s multiple comparisons test. All statistical tests were performed using GraphPad Prism 6.01 statistical package. A probability level of less than 0.05 was considered statistically significant.

## Results

### Dose-dependent effect of irbesartan on ulcer index of indomethacin-treated rats

**Figure 1:**
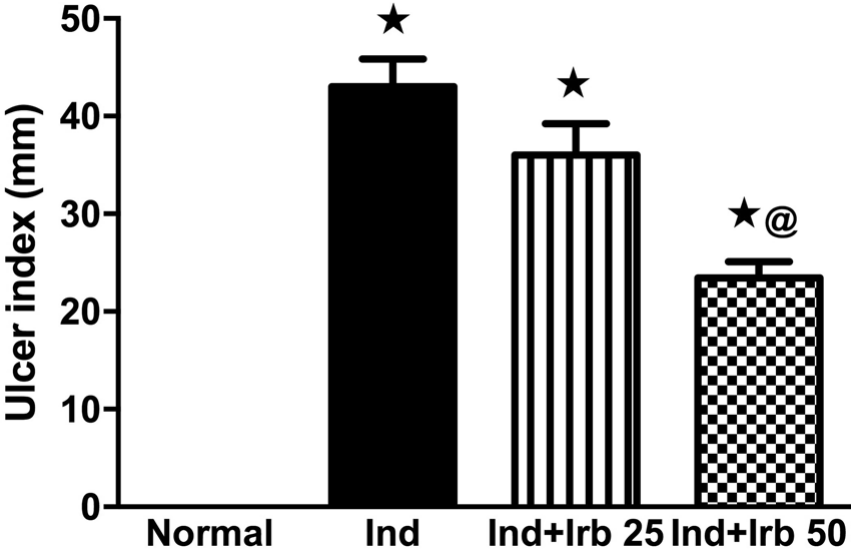
Dose-dependent effect of irbesartan on ulcer index of indomethacin ulcerated rats. Each bar with vertical line represents the mean ± S.E.M of 6 rats. *vs normal, ^**@**^vs Ind (one-way ANOVA followed by Tukey’s multiple comparisons test; p < 0.05). Irb, Irbesartan; Ind, indomethacin, Ran, ranitidine.

Treatment with Irb in a dose of 50 mg/Kg resulted in a significant reduction in ulcer index as compared to Ind-treated group (Fig. 1).

### Effect of irbesartan on gastric damage and gastric acidity alteration induced by indomethacin in rats

**Figure 2:**
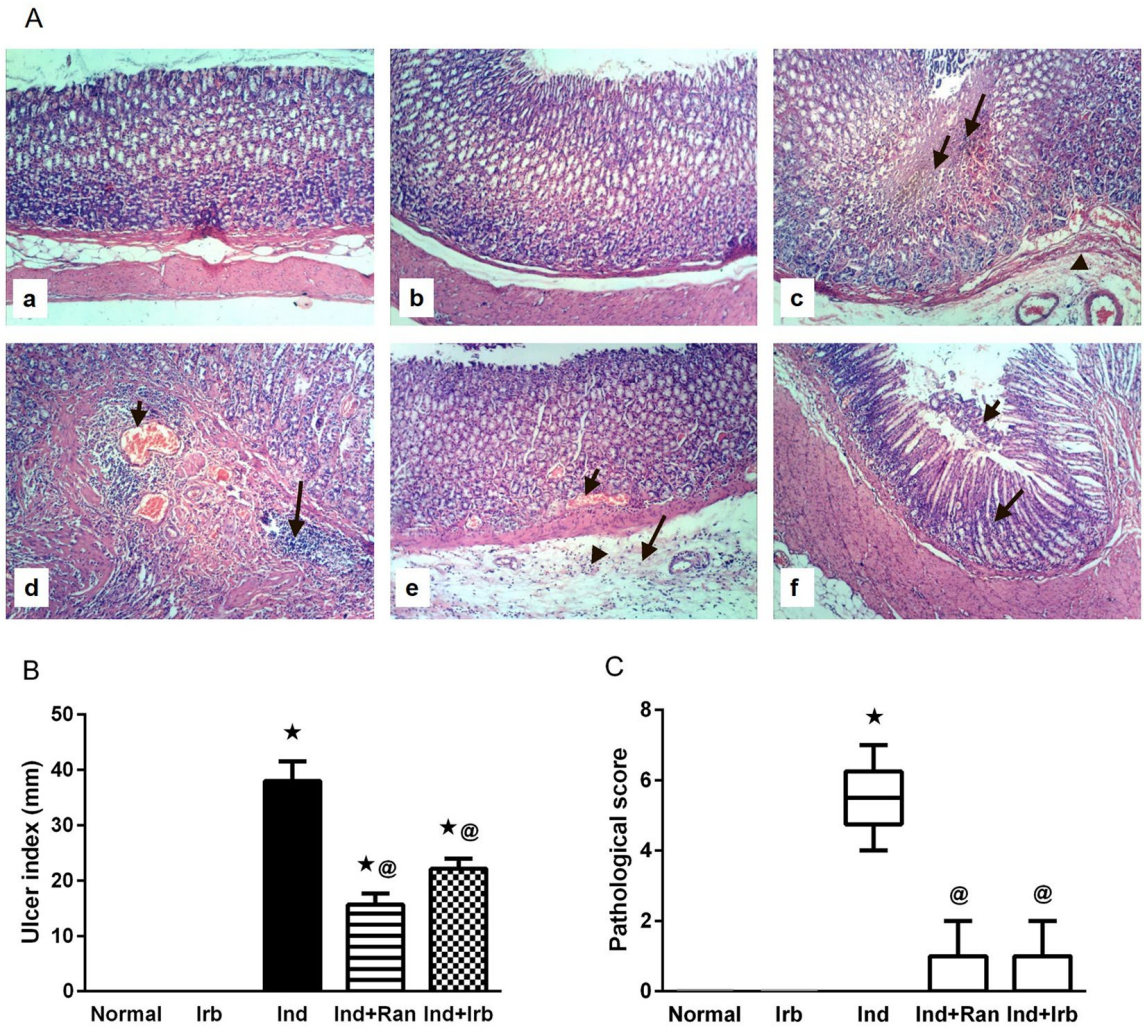
Effect of irbesartan on gastric mucosal damage induced by indomethacin in rats. (**A**) Histological assessment of gastric tissues using H&E stain (X 100). (a) Normal group showed normal histology of gastric layers. (b) Irb group showed no histopathological changes in the gastric layers. (c) Ind group showed focal coagulative necrosis of gastric mucosa (small arrow); associated with inflammatory cells infiltration (large arrow) and submucosal edema (arrow head). (d) Ind group showed congestion of submucosal blood vessels (small arrow) and massive submucosal inflammatory cells infiltration (large arrow). (e) Ind + Ran group showed mild signs of congestion of mucosal blood vessel (small arrow) and submucosal edema (large arrow) as well as few submucosal inflammatory cells infiltration (arrow head). (f) Ind + Irb group showed mild focal necrosis and sloughing of gastric mucosa (small arrow) beside infiltration of few inflammatory cells (large arrow). (**B**) Ulcer index. Each bar with vertical line represents the mean ± S.E.M of 6 rats. *vs normal, ^**@**^vs Ind (one-way ANOVA followed by Tukey’s multiple comparisons test; p < 0.05). (**C**) Pathological score. Each bar with vertical line represents the median ± range of 6 rats. ^*^vs normal, ^**@**^vs Ind (Non-parametric Kruskal-Wallis one-way ANOVA followed by Dunn’s multiple comparisons test). Irb, Irbesartan; Ind, indomethacin, Ran, ranitidine.

**Figure 3:**
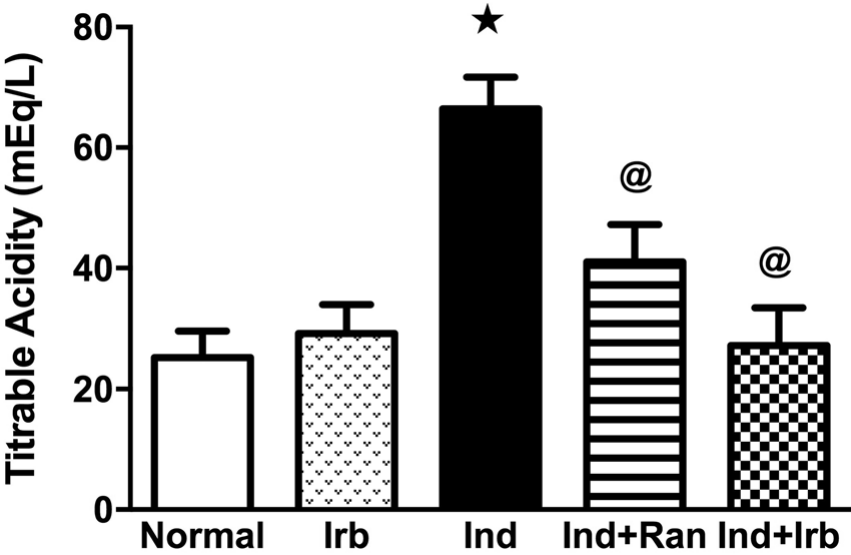
Effect of irbesartan on changes in gastric acidity induced by indomethacin in rats. Each bar with vertical line represents the mean ± S.E.M of 6 rats. *vs normal, ^**@**^vs Ind (one-way ANOVA followed by Tukey’s multiple comparisons test; p < 0.05). Irb, Irbesartan; Ind, indomethacin, Ran, ranitidine.

Exposure to indomethacin provoked a significant gastric damage in rats manifested by the pathological observation of focal coagulative necrosis of gastric mucosa, submucosal edema, and congestion of submucosal blood vessels, in addition to massive mucosal and submucosal inflammatory cells infiltration (Fig. 2A); effects that reinforce the ulcer index (Fig. 2B), pathological score (Fig. 2C), and gastric acidity (Fig. 3) results of indomethacin-treated rats. Pretreatment with ranitidine and irbesartan significantly alleviated indomethacin-induced pathological changes in the stomach and consequently decreased the pathological score as well as the ulcer index and gastric acidity. The preventive indices for ranitidine- and irbesartan-pretreated rats were 58.7 and 41.6%, respectively.

### Effect of irbesartan on changes in dimethylarginine dimethylaminohydrolase-1 expression and asymmetric dimethylarginine level induced by indomethacin in rat gastric mucosa

**Figure 4:**
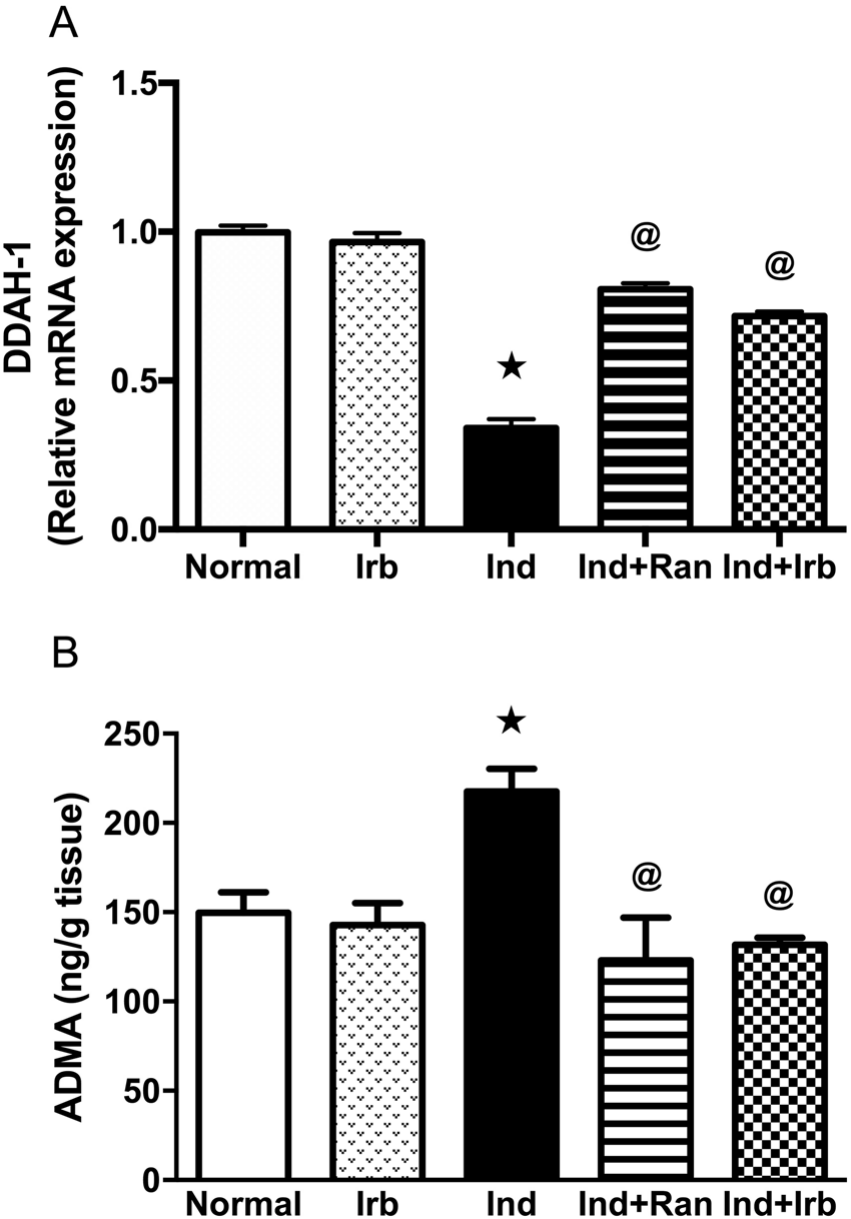
Effect of irbesartan on changes in dimethylarginine dimethylaminohydrolase-1 and asymmetric dimethylarginine level induced by indomethacin in rat gastric mucosa. (**A**) Relative mRNA expression of DDAH-1. Each bar with vertical line represents the mean fold change ± S.E.M of 6 rats compared to normal. (**B**) Level of ADMA Each bar with vertical line represents the mean ± S.E.M of 6 rats. *vs normal, ^**@**^vs Ind (one-way ANOVA followed by Tukey’s multiple comparisons test; p < 0.05). Irb, Irbesartan; Ind, indomethacin, Ran, ranitidine.

Administration of indomethacin resulted in a marked increase in ADMA level by 1.5-fold alongside a marked decrease in DDAH-1 mRNA level by 65.6% in comparison to normal rats. Both ranitidine and irbesartan pretreatment completely normalized DDAH-1 gene expression and ADMA level (Fig. 4).

### Effect of irbesartan on inflammatory changes induced by indomethacin in rat gastric mucosa

Indomethacin administration prompted a state of inflammation as demonstrated by a significant elevation of COX-2 mRNA level as well as PGE_2_, TNF-α and IL-4 levels to approximately 2-fold as compared to the normal rats. Pretreatment with ranitidine significantly reduced COX-2, PGE_2_ and TNF-α levels by 25.8, 34.4 and 47.2%, respectively, as compared to Ind group. Similarly, irbesartan significantly decreased the aforementioned inflammatory markers by 21.5, 29.6 and 37.3%, respectively, versus indomethacin-treated rats. On the other hand, IL-4 was mildly, but significantly, decreased only in ranitidine-pretreated rats by 18.5% as compared to indomethacin-treated rats (Table 3).

**Table 3:** Effect of Irbesartan on inflammatory changes induced by indomethacin in rat gastric mucosa.

Groups	COX-2 (Relative mRNA expression)	PGE-2 (ng/g tissue)	TNF-α (pg/g tissue)	IL-4 (pg/g tissue)
Normal	1 ± 0.02	10.86 ± 0.18	5.48 ± 0.07	41.72 ± 0.51
Irb	1.12 ± 0.01	10.63 ± 0.18	5.27 ± 0.03	42.25 ± 0.61
Ind	1.86 ± 0.01^★^	23.28 ± 1.48^★^	13.07 ± 1.01^★^	77.64 ± 2.56^★^
Ind + Ran	1.38 ± 0.01^★**@**^	15.27 ± 0.34^★**@**^	6.89 ± 0.09^**@**^	63.22 ± 0.73^★**@**^
Ind + Irb	1.46 ± 0.01^★**@**^	16.38 ± 0.47^★**@**^	8.19 ± 0.20^★**@**^	73.88 ± 0.67^★^

Each value represents the mean ± S.E.M. of 6 rats. *vs normal, ^**@**^vs Ind (one-way ANOVA followed by Tukey’s multiple comparisons test; p < 0.05). Irb, Irbesartan; Ind, indomethacin, Ran, ranitidine.

### Effect of irbesartan on apoptotic changes induced by indomethacin in rat gastric mucosa

**Figure 5:**
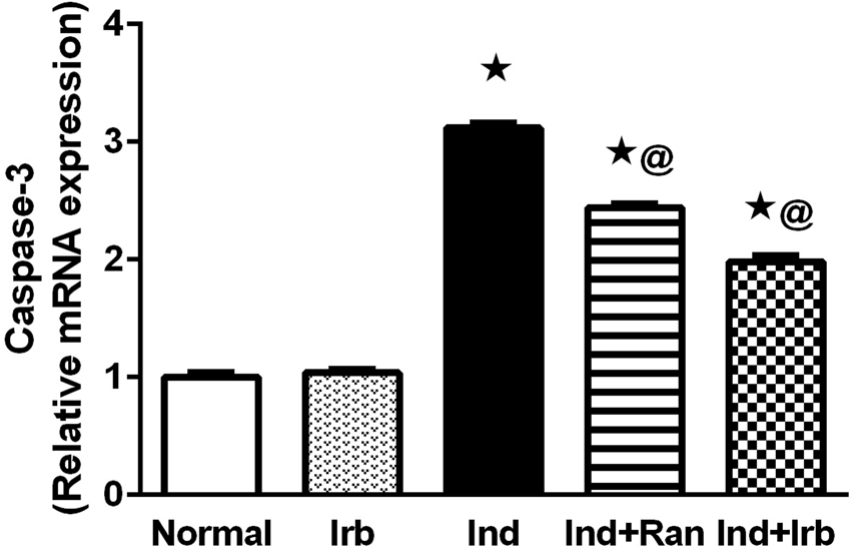
Effect of irbesartan on apoptotic changes induced by indomethacin in rat gastric mucosa. Each bar with vertical line represents the mean fold change ± S.E.M of 6 rats compared to normal. *vs normal, ^**@**^vs Ind (one-way ANOVA followed by Tukey’s multiple comparisons test; p < 0.05). Irb, Irbesartan; Ind, indomethacin, Ran, ranitidine.

Indomethacin significantly up-regulated the expression of caspase-3 at the mRNA level by 3.1-fold as compared to the normal group. On the other hand, pretreatment with ranitidine and irbesartan ameliorated such increment by 31.9 and 34.5% in comparison to indomethacin-treated rats (Fig. 5).

### Effect of irbesartan on EGFR/pERK signaling pathway changes induced by indomethacin in rat gastric mucosa

**Figure 6:**
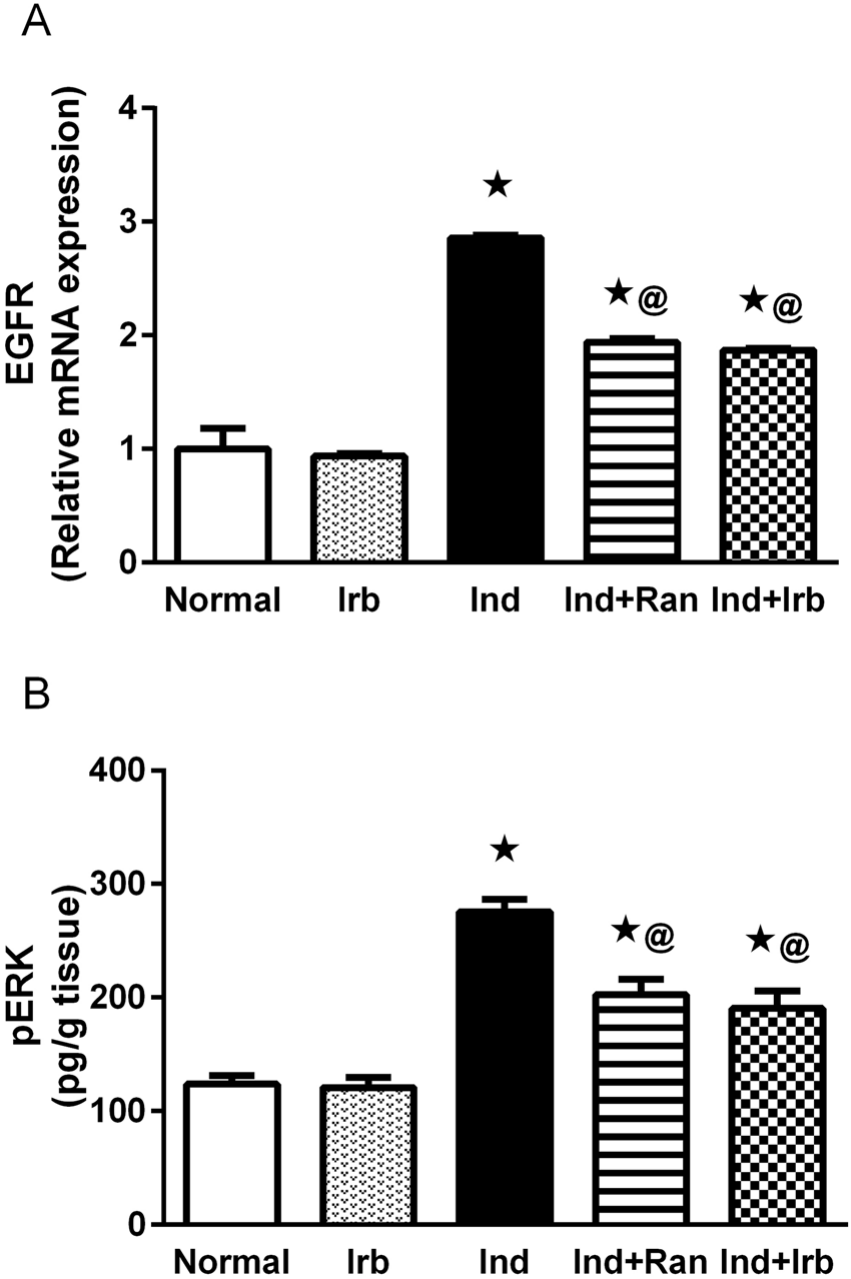
Effect of irbesartan on EGFR/pERK signaling pathway changes induced by indomethacin in rat gastric mucosa. (**A**) Relative mRNA expression of EGFR. Each bar with vertical line represents the mean fold change ± S.E.M. of 6 rats compared to normal. (**B**) Level of pERK. Each bar with vertical line represents the mean ± S.E.M of 6 rats. *vs normal, ^**@**^vs Ind (one-way ANOVA followed by Tukey’s multiple comparisons test; p < 0.05). Irb, Irbesartan; Ind, indomethacin, Ran, ranitidine.

Induction of mucosal injury markedly increased the gene expression of EGFR as well as the level of pERK to 2.8- and 2.2-fold, respectively, when compared to normal rats. Ranitidine and irbesartan pretreatment significantly attenuated the gene expression of EGFR by 32.2 and 34.6%, respectively, as well as the level of pERK by 26.5 and 30.9%, respectively, compared to Ind group (Fig. 6).

### Effect of irbesartan on changes in matrix metalloproteinase-9 expression induced by indomethacin in rat gastric mucosa

**Figure 7:**
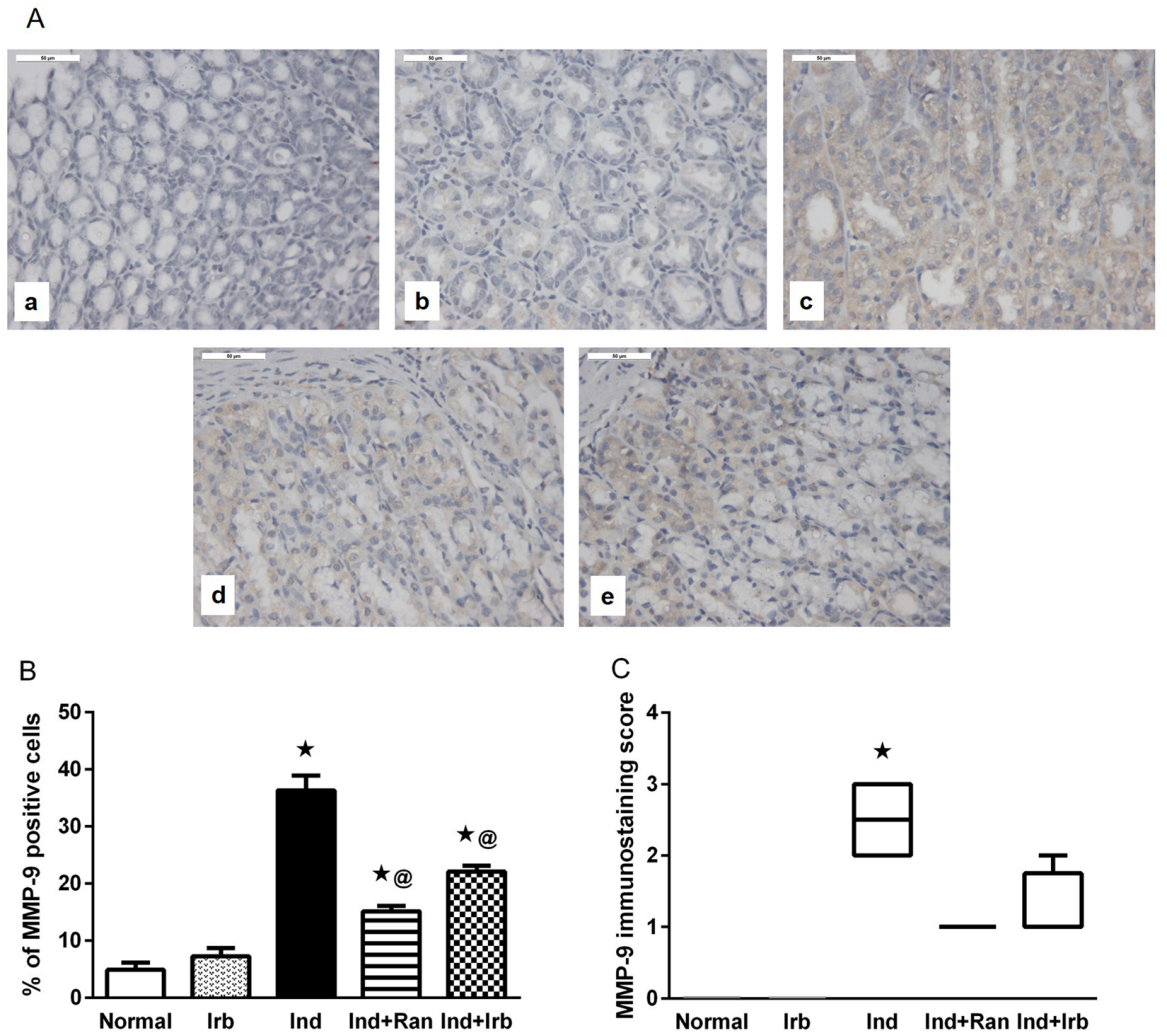
Effect of irbesartan on changes in matrix metalloproteinase-9 expression induced by indomethacin in rat gastric mucosa. (**A**) Immunohistochemical staining of MMP-9 in gastric sections (X 400). (a) Normal group showed no expression of MMP-9. (b) Irb group showed no expression of MMP-9. (c) Ind group showed strong expression of MMP-9 indicated by brown staining. (d) Ind + Ran group showed mild expression of MMP-9. (e) Ind + Irb group showed mild expression of MMP-9. (**B**) Quantification of MMP-9 staining as area percentage of immunopositive cells to the total area of microscopic field across 6 fields. Each bar with vertical line represents the mean ± S.E.M. of 6 fields. *vs normal, ^**@**^vs Ind (one-way ANOVA followed by Tukey’s multiple comparisons test; p < 0.05). (**C**) Immunostaining score of MMP-9. Each bar with vertical line represents the median ± range of 6 rats. *vs normal, ^**@**^vs Ind (Non-parametric Kruskal-Wallis one-way ANOVA followed by Dunn’s multiple comparisons test). Irb, Irbesartan; Ind, indomethacin, Ran, ranitidine.

Immunohistochemical examination of rat gastric tissues showed a significant increase in the expression of MMP-9 in indomethacin-treated rats, evidenced by the significantly higher immunohistochemical score and immunoreactive area percentage relative to intact mucosa. Pretreatment with ranitidine or irbesartan resulted in a significant suppression of MMP-9 protein expression as compared with Ind group (Fig. 7).

## Discussion

The present study depicted for the first time a significant gastroprotective potential of irbesartan in indomethacin-induced gastric injury in rats. Our findings not only extend previous reports on gastroprotective effects of other ARBs such as telmisartan, candesartan, losartan and valsartan^10,11^, but also provide new mechanistic insights into the gastroprotective role of the tested drug. Besides demonstrating acid inhibitory, anti-inflammatory and anti-apoptotic actions of irbesartan, which are in line with reported gastroprotective mechanisms of other ARBs, we also report, for the first time, a modulatory effect of an ARB, precisely irbesartan, on ECM remodeling and on DDAH/ADMA and EGFR/ERK signaling as contributing mechanisms to its gastroprotective potential.

Pretreatment with irbesartan ameliorated gastric mucosal damage as verified by a significant decrease in ulcer index and pathological score values in addition to a considerable preventive index; results that are quite comparable to those obtained by the reference drug, ranitidine. In context, an earlier study revealed an improvement of gastric emptying and gastric microcirculation by irbesartan in diabetic rats^15^.

ADMA has been considered a clinical and experimental biomarker related to gastric mucosal injury^29,31^. Promisingly, the elevated ADMA level associated with mucosal injury was reverted back to normal by irbesartan. It is well established that most of ADMA is eliminated due to the activity of DDAH-1. The observed decrease in DDAH-1 gene expression and the subsequent increase of ADMA level in injured mucosa are consistent with the report of Wang and co-workers^30^. Reduced DDAH gene expression might be related to the deranged oxidative status associated with gastric injury; a strict correlation has been suggested between the generation of ROS and ADMA^44^. A potential source of free radicals in indomethacin-injured mucosa may be the pro-oxidant activity of indomethacin resulting from peroxidase-catalyzed metabolism of NSAIDs^45^. Normalization of gastric mucosal ADMA levels in irbesartan-treated rats could be attributed to its reported ability to restore the reduced gene expression level of the main ADMA degrading enzyme, DDAH-1, through its antioxidant properties^46^. Irbesartan possesses a peroxisome proliferator-activated receptor-gamma (PPAR-γ) agonistic activity. Activation of this receptor induces catalase and superoxide dismutase gene expression, thus, mitigating oxidative stress^18,47^.

Administration of indomethacin provoked a heightened state of inflammation, as verified by significant increments in gastric mucosal TNF-α and IL-4 levels as well as COX-2 gene expression and PGE_2_ content, compared to intact gastric mucosa. Ind + Irb group exhibited a significant decline in mucosal TNF-α content and inflammatory cell infiltration indicating an anti-inflammatory role of irbesartan. Similar suppression of indomethacin-induced elevation of gastric mucosal TNF-α level was attained by telmisartan^10^. Moreover, a suppressive effect of losartan on plasma TNF-alpha level was reported in a chronic acetic acid-induced gastric ulcer model in rats^12^. In fact, TNF-*α* has been shown to be a major contributor to indomethacin-induced gastric mucosal injury^21,25^. This pro-inflammatory cytokine activates the nuclear factor kappa B (NF-κB) pathway^48^ which, in turn, promotes the transcription of a range of adhesion factors involved in neutrophil–endothelial interaction^49^, thereby accounting for the massive inflammatory cell infiltration observed in indomethacin-treated rats. Noteworthy, infiltrating inflammatory cells could be a major source of ROS generation that would further contribute to the dysregulated oxidative status^50^.

Alterations in TNF-α levels and inflammatory cell infiltration in indomethacin-treated rats and irbesartan-pretreated rats could be related to the corresponding changes in ADMA levels. Notably, it has been reported that treatment of gastric epithelial cells with exogenous ADMA resulted in a significant increase in TNF-α level^30^. Indeed, besides direct inhibition of NOS, ADMA may facilitate gastric mucosal injury as an inflammatory cytokine^51^. In a study by Kwiecien and co-workers, ADMA has been shown to aggravate stress-induced gastric injury via enhancing the overexpression and release of the proinflammatory cytokines IL-1β and TNF-α^29^. Concomitant decline in gastric mucosal TNF-α level and inflammatory cell infiltration with restoration of normal ADMA level in Ind + Irb group is in line with the reported suppressive effect of the ARB losartan on ADMA-induced increase in TNF-α level and monocyte-endothelial cell binding^8^.

The present study revealed that induction of gastric injury activated the EGFR/ERK1/2 signal transduction pathway, as manifested by significant increases in gastric mucosal mRNA expression level of EGFR and protein level of phosphorylated ERK1/2 compared to normal mucosa. Similar activation of ERK1/2 signaling has been reported in indomethacin-mediated gastric damage in mice^48,50^. Studies clearly indicate that activation of EGFR is an important early event in gastric mucosal regeneration following acute injury^27^, probably reflecting the requirement of more EGFR for ulcer healing^52^. ERK phosphorylation and activation occur in response to a variety of stimuli including elevated TNF-α^53^ and ADMA^54^, EGFR activation^28^ and oxidative stress^55^. Upon phosphorylation of ERK 1 and 2, they translocate to the nucleus and phosphorylate transcription factors, thereby triggering several cell responses^28^. Irbesartan markedly attenuated the EGFR/ERK signaling cascade triggered by indomethacin, as demonstrated by significant decrements in EGFR mRNA and pERK1/2 protein levels compared to the values of injured mucosa. Several lines of evidence support the role of EGFR/ERK1/2 inhibition in protection against gastric pathologies^50,56^. The down-regulatory effect of irbesartan on ERK1/2 phosphorylation could be attributed to its observed suppressive effect on ADMA and TNF-α levels, in addition to its reported antioxidant properties^46^.

The significantly lowered acidity shown in irbesartan pre-treated rats could be attributed to the inhibitory effect of irbesartan on ERK activation. This explanation relies on the notion that the ERK pathway may mediate H^+^, K^+^-ATPase α-subunit gene expression, contributing to gastric acid secretion in parietal cells^57^. Telmisartan was also reported to reduce elevated gastric acidity associated with indomethacin-induced ulceration^10^.

COX-2, the inducible isoform of cyclooxygenase, is a representative pro-inflammatory mediator in gastrointestinal damages^58^. The present study clearly revealed a down-regulation of COX-2 expression accompanied by a decrease in the PGE_2_ content in irbesartan-pretreated rats. In fact, irbesartan prevented angiotensin II-induced increase in COX-2 mRNA and PGE_2_ production in cultured rat mesangial cells^59^. Enhanced COX-2 and PGE_2_ expression in indomethacin-injured mucosa was previously described^58^. Additionally, based on the fact that COX-2 is the major source of prostanoid formation in inflammation^60^, our findings are also in accordance with formerly reported up-regulation of gastric COX-2 in indomethacin-treated mice^61^. Such up-regulation of COX-2 following inhibition of COX-1 by indomethacin could be regarded as a compensatory response to the inhibition of prostaglandin biosynthesis resulting from COX-1 inhibition^62^. Moreover, the up-regulated COX-2 expression in the injured mucosa could have been triggered by the elevated TNF-α level^58^ and the enhanced EGFR/ERK signaling^63^. In this respect, the suppressive effects of irbesartan on TNF-α level, EGFR expression and ERK1/2 phosphorylation in the Ind + Irb group could account for the down-regulated expression of COX-2 and the decreased production of PGE_2_.

Among the MMPs identified, MMP-9 is important in degradation of ECM and basement membrane barriers during gastric ulcer formation. It seems to play a vital role in the early phase of gastric ulcerogenesis^64^. The increase in MMP-9 expression in the injured mucosa observed herein reflects a malfunctioning of the connective tissue remodeling process^65^, and lends support to former studies^23,50,64^. On the other hand, decreased tissue staining for MMP-9 in the Ind + Irb group indicates a reduction in the proteolysis of the mucosa^66^. TNF-α, significantly elevated in this study, is one of the most important inducers of MMP production^66^. Moreover, MMP-9 expression is tightly controlled at the transcriptional level by the MAPK cascade, primarily ERK1/2, which in turn exerts its control over transcriptional factors activation through phosphorylation on the critical serine/threonine residues^43^. Therefore, the current up-regulation of MMP-9 in indomethacin-treated rats could be mediated by the observed activation of ERK1/2 signaling. The suppressive effect of irbesartan on gastric mucosal TNF-α level and ERK1/2 signaling could justify the decline in MMP-9 expression depicted in Ind + Irb rats. Furthermore, the effect of irbesartan on MMP-9 expression could be attributed to its inhibitory effect on COX-2 expression^67^.

The current study revealed a significant down-regulation of mucosal mRNA expression of caspase-3, an index of apoptotic cell death, in irbesartan-pretreated rats. An earlier study demonstrated that angiotensin II receptor blockade by telmisartan reduced the elevation of gastric mucosal caspase-3 activity in diabetic rats with indomethacin-induced gastric ulceration^10^. Enhancement of caspase-3 activation and epithelial cell apoptosis are important pathological events characterizing NSAIDs-induced gastric mucosal injury^25^. TNF-α, which was prominently elevated in the injured mucosa in the present study, and ROS, which are presumably overproduced by the infiltrating inflammatory cells, have been postulated to play a pivotal role in indomethacin-induced gastric mucosal apoptosis^10^. Moreover, administration of ADMA was reported to induce apoptosis in gastric epithelial cells^51^. Thus, the anti-apoptotic activity of irbesartan is probably related to its ability to decrease mucosal ADMA and TNF-α levels and halt the infiltration of inflammatory cells as a major source of ROS generation. Our findings that enhanced apoptosis was coupled with elevated pERK1/2 level corroborate other studies suggesting that ERK1/2 activation mediates apoptosis in gastric epithelial cells^68^ and gastric macrophages^69^. Therefore, the observed suppressive effect of irbesartan on ERK1/2 signaling might further contribute to its anti-apoptotic potential.

Altogether, this study substantiates a potent gastroprotection of irbesartan against indomethacin-induced gastric mucosal injury with comparable efficacy to ranitidine. This ARB exhibited acid inhibitory, anti-inflammatory, anti-apoptotic and ECM remodeling mechanisms that are probably mediated by suppressing DDAH/ADMA and EGFR/ERK1/2 signaling. The prospective use of irbesartan as a protective agent against gastric injury remains an area open to future investigation. Comparing irbesartan with other reportedly gastroprotective ARBs regarding their anti-ulcer capacities is an important subject that needs to be addressed. Such comparative studies may be of great value in devising optimum strategies for using ARBs in this respect.
